# 216. A Phase 1 Study of the Safety, Tolerability, and Pharmacokinetics of Multiple Doses of the Beta-lactamase inhibitor Xeruborbactam Alone and in Combination Meropenem in Healthy Adult Subjects

**DOI:** 10.1093/ofid/ofac492.294

**Published:** 2022-12-15

**Authors:** David Griffith, Jason Roberts, Steven Wallis, Maria Patricia Hernandez-Mitre, Elizabeth Morgan, Shawnee Gehrke, Michael Dudley, Jeff Loutit

**Affiliations:** Qpex Biopharma, San Diego, California; The University of Queensland, Brisbane, Queensland, Australia; University of Queensland, Brisbane, Queensland, Australia; University of Queensland, Brisbane, Queensland, Australia; Qpex Biopharma, San Diego, California; Qpex Biopharma, San Diego, California; Qpex Biopharma, San Diego, California; Qpex Biopharma, San Diego, California

## Abstract

**Background:**

Xeruborbactam (XERU) is a member of a new class of cyclic boronic acid β-lactamase inhibitors with inhibitory activity against major members of Class A, B, C, and D beta-lactamases. This report describes the safety and pharmacokinetics of XERU following multiple doses alone and in combination with meropenem.

**Methods:**

Twenty-four healthy subjects were enrolled into one of 3 cohorts with XERU alone. XERU was administered as a 500 mg loading dose followed by 250 mg q8h or 1000 mg loading dose followed by 500 mg q8h. 15 healthy subjects were enrolled in a single cohort of meropenem alone administered as a 4000 mg loading dose followed by 2000 mg q8h or meropenem in combination with XERU administered as a 500 mg loading dose followed by 250 mg q8h for 10 days. Intensive plasma sampling was obtained after dosing and assayed for drug content using validated LC-MS/MS methods.

**Results:**

XERU and Meropenem Steady State pharmacokinetic parameters are shown in the table below.

No subjects discontinued due to AEs and no SAEs were observed. There was no evidence of increasing numbers or severity of AEs with increasing dose. All AEs were mild or moderate in severity.

**Conclusion:**

XERU was safe and well-tolerated when administered alone and in combination with meropenem. XERU has unique PK properties that includes a long elimination half-life that provides sustained plasma concentrations. The loading dose of XERU allows for rapid achievement of steady state plasma levels of XERU within the first day of treatment. XERU plasma PK properties, along with a broad spectrum of inhibitory activity, facilitates its use with multiple beta-lactam antibiotics including meropenem.

**Disclosures:**

**David Griffith, n/a**, Qpex Biopharma: Employee **Jason Roberts, BPharm(Hons), PhD, FSHP, FISAC**, British Society of Chemotherapy: Grant/Research Support|Cipla: Honoraria|Gilead: Advisor/Consultant|MSD: Advisor/Consultant|MSD: Honoraria|Pfizer: Board Member|Pfizer: Honoraria|Qpex: Grant/Research Support|Sandoz: Board Member|Summit: Advisor/Consultant|Wolters Kluwer: Advisor/Consultant **Elizabeth Morgan, n/a**, Qpex Biopharma: Employee **Shawnee Gehrke, n/a**, Qpex Biopharma: Employee **Michael Dudley, n/a**, ArrePath: Board Member|Qpex Biopharma: Board Member|Qpex Biopharma: Employee **Jeff Loutit, MBChB**, Qpex Biopharma: Employee.

**Table:** 

Parameter	XERU alone	XERU alone	XERU (Combo)
	250 mg q8h	500 mg q8h	250 mg q8h
	(N=5)	(N=6)	(N=9)
C_max_ (mg/L)	69.0 ± 7.5	99.9 ± 9.7	60.7 ± 15.1
AUC_0-tau_ (mg·h/L)	472.1 ± 45.4	649.6 ± 44.4	388.4 ± 39.7
Clearance (L/h)	0.53 ± 0.06	0.77 ± 0.06	0.65 ± 0.07
Vss (L)	15.5 ± 2.6	17.8 ± 2.0	18.2 ± 1.9
Half-Life (h)	31.0 ± 1.5	24.9 ± 1.8	30.1 ± 4.9

